# Special issue: Developmental perspectives on the transition of acute to chronic pain after surgery

**DOI:** 10.1080/24740527.2022.2090323

**Published:** 2022-07-26

**Authors:** Brittany N. Rosenbloom, Maria Pavlova, Joel Katz

**Affiliations:** Child Health Evaluative Sciences, Research Institute, The Hospital for Sick Children, Toronto, CanadaBrittany.rosenbloom@sickkids.ca; Department of Psychology, University of Calgary, Calgary, Canada; Department of Psychology, York University, Toronto, Canada; Transitional Pain Service and Pain Research Unit, Department of Anesthesia and Pain Management, Toronto General Hospital, Toronto, Canada; Department of Anesthesiology and Pain Medicine, University of Toronto, Toronto, Canada

It is with great enthusiasm that we present the *Canadian Journal of Pain*’s Special Issue titled Developmental Perspectives on the Transition of Acute to Chronic Pain after Surgery. Over the past 25 years there has been an exponential growth in research on the factors involved in the transition from acute to chronic pain in infants, children, and adolescents. This Special Issue features novel advances in research and theory underlying the transition from acute to chronic pain using surgery as a model. These advances span basic, clinical, and translational perspectives that highlight a biopsychosocial framework of the development and treatment of pediatric chronic postsurgical pain (CPSP).

CPSP develops in 11% to 54% of children and adolescents after major surgery^1,2^ and significantly impacts individual functioning, including school attendance and participation in social and physical activities.^3^ The articles in this Special Issue, authored by leading international experts, make it clear that the development of pediatric CPSP is multifactorial and influenced by biological,^4–6^ psychological,^2,7^ and social^8^ factors that necessitate a multipronged approach to treatment.^6,9–11^

Using an eight-step process, Sieberg and colleagues^6^ set the stage for future research in pediatric CPSP. They promote the use of ongoing and continuous evaluation and treatment of premitigating factors including premorbid status, surgery, and immediate postsurgery treatments, as well as objective assessment of the transition to chronicity and treatment rehabilitative processes. Essential to the effective assessment and treatment of pediatric CPSP in humans is understanding the biological mechanisms underlying the transition to pain chronicity. Walker’s^5^ state-of-the-art review describes the parallels between clinical observation and translational laboratory studies that investigate the acute and long-term effects of surgical injury on nociceptive pathways. Importantly, Walker^5^ draws attention to the potential sex-dependent effects of various forms of peripheral and central neuroplasticity, including *hyperalgesic* or *nociceptive priming*, that may contribute to an increased risk of CPSP in adults scheduled for surgery who underwent a prior medical procedure or surgery in infancy or childhood. Dourson and colleagues^4^ review the current preclinical and clinical evidence for genetic and epigenetic mechanisms relevant to infant, childhood, and adolescent CPSP. They, too, focus on the complex interactions between neurons and immune cells in contributing to the phenomenon of nociceptive priming and to establishing a cellular “memory” of early life injury. Their forward-looking view anticipates the possibility of epigenetically programmed drugs to prevent pediatric CPSP.

Individual psychological processes are a key part of pediatric CPSP with psychological factors often preceding the onset of and contributing to the maintenance of CPSP.^12^ Higher levels of anxiety, depression, and parent pain catastrophizing, as well as lower levels of youth pain coping efficacy, put children and adolescents at risk of developing CPSP.^1,2,13^ Memory for pain has been posited to be one of the factors contributing to CPSP. An emerging body of research has examined the role of negatively biased pain memories (i.e., recalling more pain compared to the initial reports) in pediatric CPSP. Remembering more pain after a major surgery predicted higher pain levels 5 months postsurgery.^14^ Fear of anxiety-related physical sensations (i.e., anxiety sensitivity) and catastrophic thinking about pain predicted development of negatively biased pain memories that, in turn, are associated with worse postsurgical pain outcomes.^15^ Yet, existing research on pain memories has been focused on the accuracy, or lack thereof, of pain recall and has been limited to single-item measures. In contrast, Waisman and colleagues^7^ develop a comprehensive model of CPSP involving the autobiographical memory system and propose that an *overgeneral memory bias* is a risk factor for CPSP.^16^ Investigating overgeneral memory in youth undergoing major surgery may allow for a more nuanced examination of pain memories and their contributions to the onset and maintenance of pediatric CPSP.

Consistent with the biopsychosocial model of CPSP, and unique to pediatrics, is the role that parents play in their youth’s perioperative pain experience and functional outcomes. Both Newton-John^8^ and Rosenbloom and Katz^2^ spotlight the potential pathways through which parents and the family system contribute to the youth’s pain coping and functional outcomes. This is a novel area of research within the context of the transition from acute to chronic postsurgical pain where both Newton-John^8^ and Rosenbloom and Katz^2^ propose theoretical models worth investigating. In addition to parents, peer support, or lack thereof, can influence youth perioperative pain outcomes particularly during adolescence when peer support, and to a lesser extent parental support, becomes more influential in buffering stress.^17^ The quality of peer relationships has been associated with pain outcomes in pediatric chronic pain.^18^ It is critical for future research to examine peer relationships in the context of pediatric CPSP, including the potential of supportive peer relationships to buffer against the distress associated with CPSP.

Interventions to prevent or reduce pediatric CPSP have been urgently needed^19^ and are now emerging. The interventions target biological, physical, and/or psychological modifiable factors that contribute to the onset and maintenance of CPSP in youth. Appropriate acute perioperative pain management, ongoing multidisciplinary assessment, and acute pain clinics are among key recommended preventive strategies.^20^ Birnie and colleagues^10^ surveyed the state of pre- and postsurgical pediatric pain care in 20 health care institutions across Canada. They emphasize the ongoing need to improve current pediatric postsurgical pain care practices across Canadian health institutions including implementation and evaluation of developmentally tailored Transitional Pain Services^21^ for youth to prevent CPCP.

The ongoing COVID-19 pandemic emphasizes the critical need for virtual interventions. Murray and colleagues^11^ report the results of a single-arm pilot study evaluating the feasibility and acceptability of the first psychological intervention delivered online during the perioperative period to prevent pediatric CPSP. The intervention is grounded in the cognitive-behavioral approach and targets youth and parent anxiety, sleep, and pain coping skills. The efficacy of this feasible and acceptable intervention will be examined in a full randomized controlled trial. Pavlova and colleagues^9^ report the feasibility and acceptability of a novel memory-reframing intervention to alter youth pain memories following major surgery. The memory-reframing strategies target negatively biased pain memories and harness the malleability of pain memories by reframing distressing memories of youth perioperative experiences. Both interventions^9,11^ emphasize the critical need to engage and collaborate with patient partners in the examination and prevention of pediatric CPSP. Patient engagement has been fruitfully used in the context of pediatric chronic pain^22^ and needs to become a prominent part of pediatric CPSP research.

This Special Issue presents the most cutting-edge ideas about the transition of acute postsurgical pain to chronic pain in infants, children, and adolescents written by the world’s leading experts. Topics span the basic, clinical, and translational sciences and underscore the critical importance of adopting a biopsychosocial framework for understanding and treating pediatric chronic postsurgical pain. We hope you will be inspired by the content and join the effort to better understand, prevent, and manage this highly prevalent and debilitating condition.
